# Diphtheria resurgence in Sada'a-Yemen, 2017–2020

**DOI:** 10.1186/s12879-022-07033-x

**Published:** 2022-01-11

**Authors:** Ahmed Abdallah Al-Dar, Mutahar Al-Qassimi, Faten Hamid Ezzadeen, Mohammed Qassime, Ahmed Mohamed Al murtadha, Yasser Ghaleb

**Affiliations:** 1 Polio Surveillance National Program, Ministry of Public Health and Population, Sana’a, Yemen; 2 World Health Organization, Country Office, Sana’a, Yemen; 3 Yemen Field Epidemiology Training Program, Sana’a, Yemen

**Keywords:** Diphtheria, Outbreak, Resurgence, Immunization coverage, Yemen

## Abstract

**Background:**

Diphtheria is a contagious vaccine-preventable disease that contributes to the high morbidity and mortality among under 5 children, especially in Yemen. As a consequence of war and collapse of the health system, a fatal epidemic occurred at the end of 2017. This study aims to describe the epidemiology of diphtheria by time, place, and person and vaccination status of affected children.

**Methods:**

A study was conducted in Sada'a governorate by using accumulative line list of diphtheria from November 2017 to September 2020 at electronic Integrated Disease Early Warning System (eIDEWS). The case definition of WHO was adopted. Data was analyzed by Microsoft Excel and Epi info- version 7.2 and multivariable logistic analysis used for identifying significant associated factors.

**Results:**

747 cases were met of WHO case definition. The annual peak of cases started during week 31 and weak 49. Males were slightly more than females (51% vs 49%) and about 35% of cases involved children aged 10 to < 15 years. The overall incidence of diphtheria and case fatality rate (CFR) were 69/ 100,000 and 6.4%, respectively. The highest CFR was among age groups under 5 years 11% (*P* < 0.001) and among females was 8%. Dysphagia and swollen lymph nodes were the predominant symptoms 98%, 92%, respectively. Based on the Vaccination status, the percentage of unvaccinated and unknown were 53% and 41% respectively, with CFR 11% among cases who received one dose. Furthermore, the most case were from Sahar 40% with case fatality rate 8% and the highest CFR was significantly higher among cases in border and ongoing conflict district (*P* < 0.05).

**Conclusions:**

The findings highlight that diphtheria is still an ongoing cause of morbidity and mortality among under 5 children in Sada'a that is rising with the low diphtheria immunization coverage. Therefore, concomitant efforts should now focus on improving and monitoring routine immunization across all age groups and healthcare services, especially in borders and continuing conflict districts.

## Introduction

Diphtheria is a highly contagious bacterial disease of upper respiratory tract and caused by *Corynebacterium diphtheriae*. It is a vaccine-preventable disease which transmitted by direct contact with droplets from an infected person’s cough or sneeze and it presents as a membranous pharyngitis. Sore throat, fever, and swollen glands in the neck are the common symptoms of diphtheria [1].

Diphtheria remains a health problem in different countries with weak vaccination coverage or pockets of unimmunized. However, after the start of the vaccine in the United Kingdom and then worldwide in the 1940–50 s [2], diphtheria was practically eliminated and clinical diphtheria became an uncommon disease in the world. Nevertheless, there is presently global interest that diphtheria is re-emerging. A number of diphtheria outbreaks have been reported from different regions in Europe [3], Southeast Asia, America [4], and Africa [5] and the risk increases among children who are unvaccinated or partially vaccinated [6].

Diphtheria has represented a major health issue in Yemen since 2017. The current situation has reached a new level of complexity, particularly after the war and conflict started in late March 2015 [7], which has critically affected health system and basic health services [8, 9]. Between October 2017 and August 2018, the surveillance system detected 2203 probable cases of diphtheria, including 116 deaths. Unfortunately, before the outbreak was declared, there were just a few diphtheria case alerts by the early warning electronic surveillance system [9].

Diphtheria outbreaks have reflected a large gap in vaccine coverage in the previous three years, due to the noticeable collapse of the health system. Furthermore, in both 2017 and 2018, most governorates achieved < 80% immunization coverage for the third dose of pentavalent vaccine, which is considered insufficient to ensure population protection [10].

This study aims to describe the epidemiology of diphtheria by time, place, and person and vaccination status of affected children.

## Methods

### Study design, population and area

Retrospective descriptive cross-sectional study was conducted in Sada'a governorate by used accumulative line list of diphtheria from 2017 to August 2020 at electronic Integrated Disease Early Warning System (eIDEWS) [11, 12]. Sada'a governorate is located in the north of Yemen and consists of fifteen districts as well as it is the site of several ongoing and varied types of conflict. In 2017, the governorate population size is 1,078,000 with 56 per km^2^ of density.

#### Diphtheria case definitions

Probable cases were defined as any person with illness characterized by an adherent membrane on the tonsils, pharynx and/or nose and any one of the following: laryngitis, pharyngitis or tonsillitis based on clinical examination.

A confirmed case was defined as a probable case that was laboratory confirmed or linked epidemiologically to a laboratory-confirmed case [13].

### Data collection

The collected data contain demographic variables and clinical information: e.g. patient identification number, sex, age, current address of patients, date of onset of symptoms, date of reporting to the health facility, signs and symptoms, treatment and clinical outcome.

The data was collected by focal point in forty nine health facilities, community surveillance coordinator and rapid response team using eIDEWS system and the data was reviewed and cleaned in the central level.

### Data analysis

Total Population of governorate was obtained from Yemen Central Statistical Organization and used to calculate Incidence rate/100,000 and the case fatality rate (CFR) per age group was calculated by dividing the number of deaths of each age group by the number of cases within each age group.

Data was analyzed by using Epi info 7.2 version where it was presented as frequencies and percentage. Cross-tabulation and multivariate logistic regression was used to identify associated factors with the mortality using the Chi-squared test (χ^2^) and odd ratio (OR). P-value of less than 0.05 at confidence interval 95% will be considered statistically significant.

## Results

**Fig. 1:**
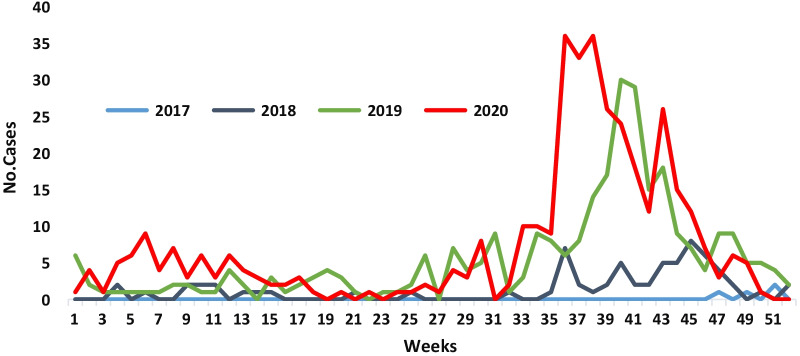
Distribution of diphtheria cases by week in sada'a, 2017–2020, Yemen (n = 747)

A total of 747 cases were reported by rapid response team in Sada'a governorate during the period November 2017 to December 2020. Figure 1 shows the distribution of diphtheria cases by epidemiological weeks. Gradual increasing in diphtheria cases from 2017 with the peak occurring in epidemiological week 36 in 2020 and decreased until 49 weak.

Table 1 shows the distribution of diphtheria case-fatality ratios by selected characteristics. Males were slightly more than females (51% vs 49%) and increase of CFR among females 8%. About 35% of cases were occurred among age group (10– < 15) as well as increase of CFR in children under 5 years old 11%. Dysphagia and swollen of lymph nodes were the predominant symptoms 94%, 92%, respectively. However, the CFR more among cases with difficulty of breathing 12%. Based on the Vaccination status, the percentage of unvaccinated and unknown were 53% and 41% respectively, with CFR 11% among case who received one dose. Bivariate analysis identified the fatality rate significantly increase among cases with age group < 5 years and had difficulty in breathing [*P* < 0.001].

**Table 1:** Diphtheria case-fatality ratios by selected characteristics in Sada'a, 2017–2020, Yemen

Variable	Reported cases n = 747		Deaths n = 48		CFR (%)	OR (95% CI)	*P* value
	Frequency	Percent	Frequency	Percent			
Sex							
Male	367	49	18	38	5	Ref	
Female	380	51	30	63	8	0.6 (0.91–3.09)	0.129
Age groups							
< 5 years	131	18	14	29	11	9.28 (1.2–71.53)	< 0.001
5–< 10 years	195	26	17	35	9	3.61 (0.47–27.93)	0.322
10–< 15 years	258	35	11	23	4	2.49 (0.31–19.88)	0.617
15–< 20 years	45	6	1	2	2	Ref	
20–< 25 years	32	4	2	4	6	1.28 (0.08–21.15)	1.00
25–< 30 years	33	4	1	2	3	1.43(0.07–23.62)	1.00
30–< 35 years	15	2	1	2	7	5.55 (0.47–65.04)	0.189
> 35 years	32	4	1	2	3	2.77 (0.24–31.81)	0.573
Unknown	6	1	0	0	0	N/A	
Signs and symptoms							
Dysphagia							
Yes	695	94	44	92	69	0.744 (0.256–2.16)	0.809
No	48	6	4	4	8	Ref	
Swollen L&N							
Yes	686	92	47	98	69	4.339 (0.59–32.01)	0.195
No	61	8	1	1	2	Ref	
Difficulty of Breathing							
Yes	290	39	32	67	11	3.403 (1.83–6.32)	< 0.001
No	455	61	16	33	7	Ref	
Vaccination status							
0 dose (Unvaccinated)	397	53	30	63	8	Ref	
1 dose	28	4	3	6	11	1.58 (0.45–5.56)	0.185
2 doses	8	1	0	0	0	N/A	
3 doses	8	1	0	0	0	N/A	
> 3 doses	2	0	0	0	0	N/A	
Unknown	304	41	15	31	5	0.68 (0.36–1.30)	0.32

Table 2 presents the distribution of diphtheria cases with case fatality rate by districts. The overall incidence of diphtheria was 69 per 100,000 population and the CFR was 6.7. The highest percentage of cases in Sahar 40% with case fatality rate 8% and the highest case fatality rate was in Monabbih 31% as well as the fatality rate significantly increase in border and ongoing conflict districts [P < 0.05]. Similar results of the associated risk factors obtained by multivariate analysis.

**Table 2:** Distribution of diphtheria cases with case fatality rate by districts in Sada'a, 2017–2020, Yemen

Districts	Population	Probable cases	Percent	Incidence rate /100000	Deaths	CFR (%)	OR (95% CI)	*P* value
Sa'ada	89,029	79	11	89	2	3	Ref	
Sahar	207,208	296	40	143	23	8	3.24 (0.78–14.04)	0.128
Majz	106,420	110	15	103	3	3	1.08 (0.18–6.61)	1.00
As Safra	78,828	75	10	95	7	9	0.40 (0.79–19.72)	0.092
Haydan	94,143	65	9	69	2	3	1.22 (0.17–8.92)	1.00
Sageen^a^	81,755	28	4	34	4	14	6.41 (1.11–37.2)	< 0.05
kitaf wa alboque	67,169	26	3	39	3	12	5.02 (0.79–31.9)	0.096
Monabbih^a^	81,002	13	2	16	4	31	17.11(2.74–106.9)	< 0.05
Qatabir	35,268	12	2	34	0	0	N/A	N/A
Al Hashwa	22,412	10	1	45	0	0	N/A	N/A
Razh	97,836	7	1	7	0	0	N/A	N/A
Gammer	30,658	4	1	13	0	0	N/A	N/A
Al Dhaher	33,710	1	0	3	0	0	N/A	N/A
Sheda	16,696	1	0	6	0	0	N/A	N/A
Bagim	35,869	20	3	56	0	0	N/A	N/A
Total	1,078,000	747	100	69	48	6.4		

^a^Border districts and ongoing conflict

## Discussion

Diphtheria re-emerging after more than 30 years, and lead to increase of motility and morbidity among children in Yemen. Due to the completely destruction and collapse of health system, diphtheria occurred as a fatal epidemic since the end of 2017. The diphtheria outbreak in Yemen developed in three epidemic waves, which affected nearly all governorates of Yemen, From November 2017 to March 2018, a mass vaccination campaign targeted nearly 2.7 million children aged 6 weeks to 15 years in 11 governorates and in 2019, diphtheria vaccination was further conducted in 186 districts of the 12 Northern governorates [14, 15].

Our study showed the increase of cases at the end of summer, this result is quite similar with previous reports in Dibrugarh district, Assam and Nigeria [16, 17].

Regarding the most affected age group, we found that the 5–15 years was the most affected which is similar to studies that reported a shift in the age group affected by diphtheria to older children and adults [18, 19]. However, this finding is in disagreement with studies in Yemen, India, and Bangladesh which found the more affected children were < 5 years [13, 20, 21]. In addition, our findings showed the children with age < 5 years had the highest CFR which is similar to previous results in Yemen [22]. This could be related to the immunization status of the target group and inadequate access to health care due to conflict and war.

Despite the fact that the Expanded Program on Immunization (EPI) provides free immunizations for children in Yemen, vaccine-preventable diseases still account for nearly one-third of all deaths among children under the age of five. Yemen had consistent vaccination coverage before 2015, reaching %70 to 80% of the target population, however, this fell significantly dropped since the beginning of the conflict [23]. Furthermore, according to the EPI report in 2020, the vaccination coverage was low at 58%, and vaccination against diphtheria in selected districts of Southern governorates and Sada’a started in July 2020 [24, 25]. Low childhood vaccination rates, and a lack of booster immunizations for older children and adults, are the common causes of diphtheria epidemics [19], this is consistent with the result of this study which showed most affected cases were unvaccinated.

In our finding, increase of cases fatality rate of females compared to males, similar result of previous studies in Banaskantha District, Gujarat and India [26, 27].This attributed to low vaccination coverage in previous among female in Yemen.

In addition, the findings showed varied incident rate and CFR of different districts, for example, the AR Sahar district 143/100,000 and Al Dhaher district 3/100,000. This attributed to, the conflict has led to major movement of population from district to others especially in border district of sada'a governorate. According to WHO risk assessment, respiratory diphtheria is fatal in 5–10% of cases, with a higher mortality rate in young children [28]. Furthermore, the overall of CFR among cases was 6.4%, this similar with study from India and slightly higher than reported in previous study in Yemen [27, 29].

In conclusion, the majority of patients were from Sahar district. The outbreak in this area could be due to the existence of pockets of low immunization coverage. Children under five years were more affected with higher fatality among ≤ 15 years. Strengthen outreach immunization coverage and introduce booster vaccination against diphtheria in whole governorate especially in difficult access district. Furthermore, increase public health awareness toward diphtheria disease to control and prevent more cases. Strengthen the surveillance for early detection, immediate response and providing antitoxin in difficult access areas are recommended.

The limitations of this study were the quality of data and under reported cases as well as the data collector have depended on patients or relatives to recall information regarding the vaccination status, which has a high probability of recall bias.

## Data Availability

The data presented in this paper are available from the corresponding author on request. In addition, the data was publish by eIDEWS program at MoPHP as weekly epidemiological bulletin.

## Abbreviations

CFR: Case Fatality Rate
EPI: Expanded Program on Immunization
eIDEWS: Electronic Integrated Disease Early Warning System
MoPHP: Ministry of Public Health and Population
NCPHL: National Centre of the Public Health Laboratories
WHO: World Health Organization

## References

[1] WHO. Diphtheria Geneva: World Health Organization 2018. http://www.who.int/immunization/monitoring_surveillance/burden/diphtheria/en/. Accessed 18 Mar 2018.

[2] Group DGW (2015). Public health control and management of diphtheria.

[3] Wagner KS, White JM, Lucenko I (2012). Diphtheria in the postepidemic period, Europe, 2000–2009. Emerg Infect Dis.

[4] Santos LS, Sant'anna LO, Ramos JN (2015). Diphtheria outbreak in Maranhao, Brazil: microbiological, clinical and epidemiological aspects. Epidemiol Infect.

[5] Besa NC, Coldiron ME, Bakri A (2014). Diphtheria outbreak with high mortality in northeastern Nigeria. Epidemiol Infect.

[6] WHO (2006). Diphtheria vaccine. Weekly Epidemiol Record..

[7] European Centre for Disease Prevention and Control. Annual Epidemiological Report–Diphtheria. Stockholm: ECDC; 2016. https://ecdc.europa.eu/en/publications-data/diphtheriaannual-epidemiological-report-2016-2014-data#copy-to-clipboard. Accessed 18 Mar 2018.

[8] WHO, MOPH&P. Weekly Epidemiological Bulletin. Epi week 32, Vol 06. Yemen: Ministry of Health. 2018; 2018:06–12.

[9] OCHA-Yemen. Humanitarian needs overview Yemen: UNOCHA. 2018.

[10] Blumberg LH, Prieto MA, Diaz JV, Blanco MJ, Valle B (2018). The preventable tragedy of diphtheria in the 21st century. Int J Infect Dis 3 IJID3..

[11] Dureab F, Ahmed K, Beiersmann C, Standley CJ, Alwaleedi A, Jahn A (2020). Assessment of electronic disease early warning system for improved disease surveillance and outbreak response in Yemen. BMC Public Health.

[12] Mayad M, Alyusfi R, Assabri A, Khader Y (2019). An electronic disease early warning system in Sana’a Governorate, Yemen: evaluation study. JMIR Public Health Surveill.

[13] WHO-recommended standards for surveillance of selected vaccine preventable diseases. World Health Organization. 1999. https://apps.who.int/iris/handle/10665/64165.

[14] OCHA-services. Diphtheria vaccination campaign for 2.7 million children concludes in Yemen. 2018. https://reliefweb.int/report/yemen/diphtheria-vaccination-campaign-27-million-childrenconcludes-yemen.

[15] WHO-EMRO. Situation Report, September 2019, Issue NO.9: Yemen Conflict. 2019. https://reliefweb.int/sites/reliefweb.int/files/resources/Yem-Sitrep-Sept-2019.pdf.

[16] Nath B, Mahanta TG (2010). Investigation of an outbreak of diphtheria in Borborooah block of Dibrugarh district, Assam. Indian J Commun Med.

[17] Besa NC, Coldiron ME, Bakri A, Raji A, Nsuami MJ, Rousseau C (2014). Diphtheria outbreak with high mortality in northeastern Nigeria. Epidemiol Infect.

[18] Clarke KEN. Review of the epidemiology of diphtheria—2000–2016. World Health Organization. 2017.

[19] Galazka A (2000). The changing epidemiology of diphtheria in the vaccine era. J Infect Dis.

[20] Jone E, Kim-farley J, Algunid M, Ballady Y (1985). Diphtheria: a possible foodborne outbreak in Hodeida, Yemen Arab Republic. Bull World Health Organization..

[21] Rahman MR, Islam K. Massive diphtheria outbreak among Rohingya refugee: lesson learnt. J Travel Med. 2018 10.1093/jtm/tay122 30407562

[22] Dureab F, Al-Sakkaf M, Ismail O, Kuunibe N, Krisam J (2019). Diphtheria outbreak in Yemen: impact of conflict on a fragile health system. Confl Health.

[23] Qirbi N, Ismail SA (2016). Ongoing threat of a large-scale measles outbreak in Yemen. Lancet Global Health.

[24] EPI-Yemen. Annual accumulative report of the Immunization coverage 2017. National Expanded program of Immunization. 2018.

[25] Badell E, Alharazi A, Criscuolo A, et al. Epidemiological, clinical and genomic insights into the ongoing diphtheria outbreak in Yemen. medRxiv. 2020. 10.1101/2020.07.21.20159186/. Accessed 10 July 2021

[26] Kadri AM, Dave BB, Desai BR (2019). Epidemic investigation of diphtheria outbreak in Banaskantha District, Gujarat. Natl J Commun Med.

[27] Murhekar M, Bitragunta S (2011). Persistence of diphtheria in India. Indian J Commun Med.

[28] WHO, Diphtheria- Yemen report. 2017. https://www.who.int/emergencies/disease-outbreak-news/item/22-december-2017-diphtheria-yemen-en.

[29] Moghalles SA, Aboasba BA, Alamad MA, Khader YS (2021). Epidemiology of diphtheria in Yemen, 2017–2018: surveillance data analysis. JMIR Public Health Surveill.

